# Microglial process convergence on axonal segments in health and disease

**DOI:** 10.20517/2347-8659.2019.28

**Published:** 2020-03-21

**Authors:** Savannah D. Benusa, Audrey D. Lafrenaye

**Affiliations:** Department of Anatomy and Neurobiology, Virginia Commonwealth University, Richmond, VA 23298, USA.

**Keywords:** Microglia, multiple sclerosis, traumatic brain injury, microglia-axonal interactions

## Abstract

Microglia dynamically interact with neurons influencing the development, structure, and function of neuronal networks. Recent studies suggest microglia may also influence neuronal activity by physically interacting with axonal domains responsible for action potential initiation and propagation. However, the nature of these microglial process interactions is not well understood. Microglial-axonal contacts are present early in development and persist through adulthood, implicating microglial interactions in the regulation of axonal integrity in both the developing and mature central nervous system. Moreover, changes in microglial-axonal contact have been described in disease states such as multiple sclerosis (MS) and traumatic brain injury (TBI). Depending on the disease state, there are increased associations with specific axonal segments. In MS, there is enhanced contact with the axon initial segment and node of Ranvier, while, in TBI, microglia alter interactions with axons at the site of injury, as well as at the axon initial segment. In this article, we review the interactions of microglial processes with axonal segments, analyzing their associations with various axonal domains and how these interactions may differ between MS and TBI. Furthermore, we discuss potential functional consequences and molecular mechanisms of these interactions and how these may differ among various types of microglial-axonal interactions.

## INTRODUCTION

Microglia are the innate immune cells of the central nervous system (CNS) and the primary mediators of the neuroinflammatory response. They are derived from a pool of primitive macrophages from the yolk sac that appear during early embryonic development^[1–3]^. Microglia are ontogenetically distinct from the peripheral blood-derived monocytes/macrophages that reside outside the CNS and mediate the peripheral inflammatory response^[4,5]^. Peripheral blood-derived immune cells are not typically found in the healthy CNS. However, peripheral monocytes/macrophages can infiltrate the CNS and exacerbate the neuroinflammatory response under pathological conditions. The distinct developmental origin of microglia from peripheral monocyte-derived macrophages and the exclusion of peripheral immune cells from the CNS underscores the immunological privilege of the CNS and the unique functions microglia might exert in the healthy brain and in pathological processes^[6]^.

Microglia are cells with highly dynamic process networks that rapidly remodel to survey the microenvironment and maintain tissue homeostasis^[7–9]^. The surveying processes of microglia respond to CNS perturbations through rapid protrusion onto the site of insult/interest^[7,10]^ and microglia undergo “activation”, a complex series of alterations including changes in enzyme, receptor, and immune factor expression and altered cellular morphology^[3,11,12]^. Microglia exhibit a variety of morphologies ranging from small cell bodies with long highly-branched processes to enlarged cell bodies with short, thick processes^[3,13]^. The spectrum of microglial morphologies is indicative of their activation state and is commonly used to characterize activated *vs.* non-activated microglia in histological samples. Surveying (non-activated) microglia exhibit long, highly-branched or “ramified” processes that sample the surrounding environment. However, upon activation, microglia retract their processes and increase their cell body size, exhibiting morphologies defined by short, thick processes and large somas^[3,14]^. Highly activated, phagocytic microglia tend to lose distinctive processes all together and exhibit an ameboid shape^[3,14]^.

Many studies have investigated microglial-neuronal interactions via secreted factors. Activated microglia exhibit extensive changes in the expression of their inflammatory profile^[15]^. While some of these secreted factors may provide neurotrophic functions, pro-inflammatory factors exhibit deleterious effects^[16,17]^. Various neurotrophic secreted factors released from microglia induce neurite outgrowth and have been shown to be involved in regulating the cytoarchitecture of the developing brain^[18–20]^. Pro-inflammatory microglia, however, up-regulate cytokines and enzymes that produce reactive oxygen species, which have been implicated in axonal injury and disruption^[16,21–32]^.

**Figure 1.**
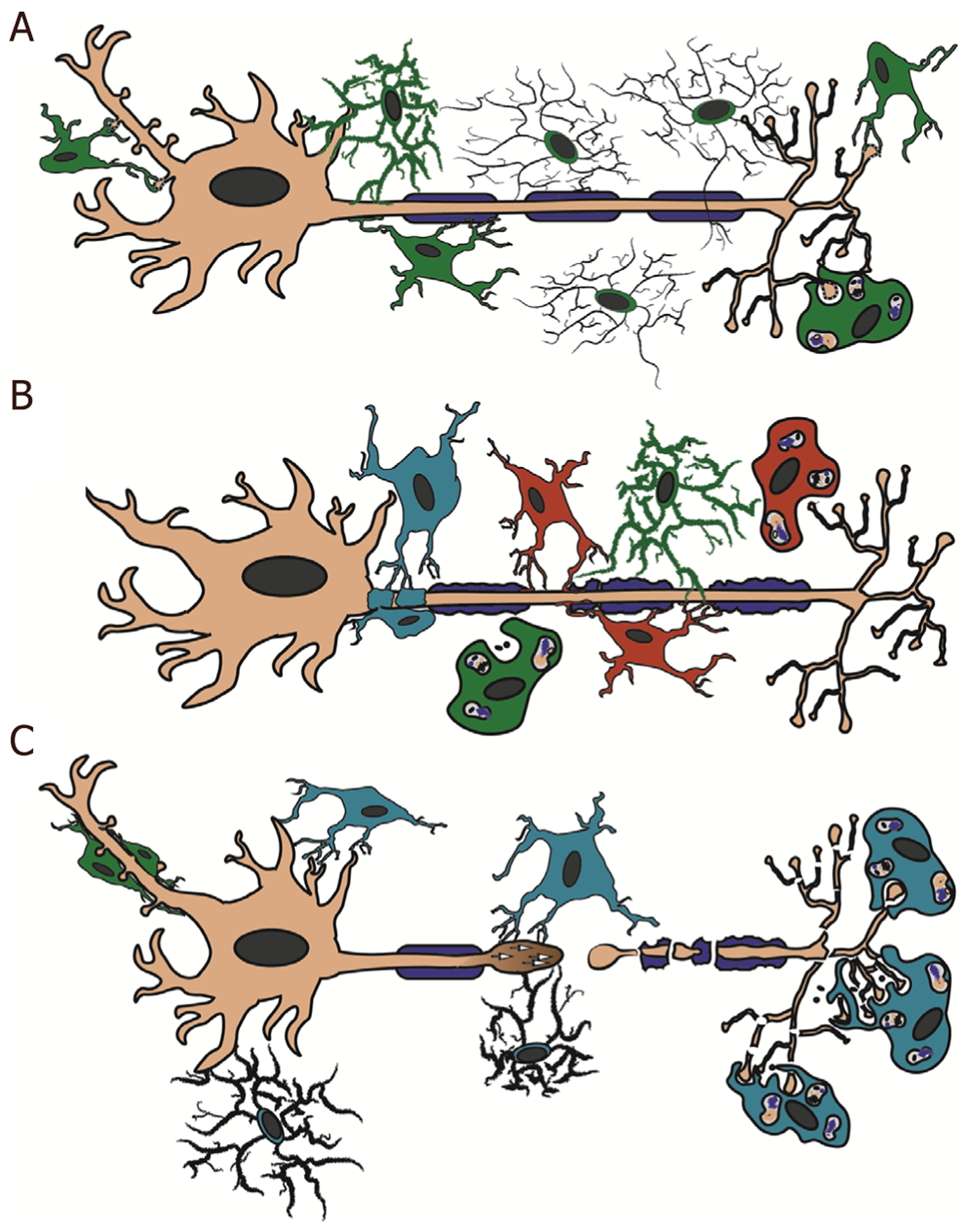
Schematic representation of microglial process contacts in health and disease. Illustration demonstrating various microglial and monocytic contacts onto axonal segments. A: in the healthy brain, resident microglia (green) contact the neuronal cell body and axon initial segment. These microglia potentially express TNF-α and CSF1 and are involved in reduction of hyperexcitability in neurons. The dynamic surveying processes of non-activated ramified microglia also contact various areas of the axon in the healthy CNS. During development, contacts by resident microglia are involved in pre- and postsynaptic pruning; B: in MS, both resident microglia (green) and infiltrating peripheral monocytes (red) contact the nodes of Ranvier. Note that the processes of monocytes are found between the layers of myelin and the axon sheath, while the resident microglial processes are primarily in contact with adjacent monocytes and/or involved in debris clearance. Neuroinflammatory cells that have yet to be identified as either resident microglia or infiltrating monocytes (teal) that express TNF-α, INOS, Nox2, and higher levels of activated calpain, wrap the axon initial segment. This wrapping is involved in a notable reduction in the length of the axon initial segment; C: following TBI, macrophages (monocytes and/or microglia) phagocytosis the Wallerian debris from the degenerating distal axonal segments of an injured axons. Potential hyperexcitability of neurons following TBI induces microglial process convergence onto the neuronal soma via elevated ATP levels and/or glutamate levels. Rod microglia (green) are also common along the apical dendrite following injury; however, their function is currently unknown. Microglial process convergence onto the proximal injured axonal segment is associated with P2Y12 and potentially confers neuroprotective effects on the damaged axon leading to axonal sprouting. CNS: central nervous system; MS: multiple sclerosis; TBI: traumatic brain injury; TNF: tumor necrosis factor; CSF1: colony stimulating factor 1; INOS: inducible nitric oxide synthase; Nox2: NADPH oxidase 2

Microglia also interact with neurons through physical contact under homeostatic conditions^[7,9,11,33–36]^. Microglia have recently been shown to contact dendrites and neuronal cell bodies in the normal adult brain^[37,38]^. Both contact types require purinergic signaling through the P2Y12 receptor and appear to be protective in nature^[37–40]^. In the developing somatosensory cortex, it was recently found that microglial process contacts onto dendrites precipitates filipodia formation, linking microglia process contacts with synaptic formation^[38]^. Microglia are also key mediators of synaptic pruning, which alters the neuronal excitatory/inhibitory balance^[41]^. Microglia contact pre- and postsynaptic neuronal elements in an activity-dependent manner, and synapses that are contacted by microglia more frequently and for longer durations of time are subsequently removed [Figure 1A]^[9,42,43]^. Specifically, studies have demonstrated that early during development (Postnatal Day 5 in mice) phagocytic microglia engulf synapses of neurons with reduced activity/input in a complement-dependent manor^[42,43]^. Alternatively, later during development (Postnatal Day 15 in mice) microglia only appear to remove parts of synapses in a process called “trogocytosis”^[44]^. Another study using zebrafish larva demonstrated that microglial-synaptic contacts increased with increased neuronal spontaneous activity. Further, the zebrafish neurons that were contacted by microglia exhibited a decrease in activity, while noncontacted neurons maintained an increased firing rate^[36]^.

Microglia may also influence neuronal excitability through contact with the axon initial segment (AIS), the axonal domain responsible for action potential initiation and modulation [Figure 1A]^[45]^. Microglia appear to establish contact with the AIS early in development and maintain this contact through adulthood, strongly suggesting that microglia play a role in regulating AIS structure and function^[45]^. Additionally, when repeated stimulations were used to induce neuronal hyperexcitability, microglia extended their processes and wrapped around axons^[35]^. This induced a rapid repolarization in the neuron back to resting levels which was lost when microglia were pharmacologically blocked^[35]^.

Neuroinflammatory microglial changes are associated with various pathologies, including, but not limited to, spinal cord injury, neurodegenerative diseases, and early-life stress^[46–49]^. Alterations in the form and frequency of physical microglial-axonal contacts, however, have been described in the most detail in multiple sclerosis (MS) and traumatic brain injury (TBI), therefore this review focuses on these two disease states^[45,50–52]^. Many of the changes in microglial-neuronal contacts appear to be dependent on the disease state, in which there are alterations in microglial associations with specific axonal segments. Below, we review the interactions of microglial processes with axonal segments, focusing on their associations with various axonal domains and the unique alterations of these physical interactions in MS and TBI.

## NEUROINFLAMMATION IN MS

MS is an autoimmune-mediated disease of the CNS that is characterized by inflammation and demyelination. While the cause of MS is not fully understood, it is accepted that neuroinflammation, resulting from the accumulation and activation of macrophages (derived from microglia or infiltrating monocytes) in the human CNS, is a crucial step in MS pathogenesis, which culminates in injury to myelin and axons and disrupts the flow of information^[53–56]^. The autoimmune nature of MS and the role of autoreactive peripheral T cells is highly complex and has been reviewed previously^[57]^. Therefore, we do not discuss the autoreactive peripheral immune cells in this review. Furthermore, destruction of myelin and axons, as well as oligodendrocyte cell-death, are directly related to the numbers of activated inflammatory cells^[53,58–60]^. The symptoms of MS range widely based on the CNS region affected and include a variety of motor or sensory dysfunctions such as muscle weakness, spasticity, tremor, unexplained pain or numbness, vision problems, and cognitive deficits^[61]^. While demyelination is a hallmark of MS, axonal injury is also a prominent pathological feature and is a major contributor of chronic disability in patients^[59,60,62–64]^. The types of axonal injuries in MS and its models include the formation of axonal swellings, reduced levels of Na^+^/K^+^ ATPase, synaptic damage, axon transection, and disruption of axonal domains, such as the node of Ranvier (NOR) and the AIS^[65–69]^. These axonal injuries may occur as either a consequence of demyelination^[65,70]^ or as a primary event, independent of myelin loss^[27,71]^, although the mechanisms driving primary axonal pathology are not fully understood. It is appreciated that soluble factors produced by resident microglia and infiltrating monocytes and their interactions with peripheral immune cells play a pivotal role in driving axonal injury^[59,60,72–75]^; however, recent studies have implicated a mechanistic role for microglia/monocytes through physical interactions with axonal domains^[6,27,28]^.

Studies investigating axonal contact by microglia and/or infiltrating monocytes have utilized two common models of MS: a toxin-induced demyelinating model, cuprizone^[27]^, and an immune-mediated model, experimental autoimmune/allergic encephalomyelitis (EAE)^[6,27]^. In the cuprizone model, a copper-chelating toxin, cuprizone, is administered through chow resulting in oligodendrocyte cell death and, consequently, loss of myelin^[75]^. Demyelination is detectable 1–2 weeks after cuprizone treatment with peak demyelination occurring by 5–6 weeks of exposure^[76–78]^. The cuprizone model yields substantial demyelination and, upon removal of toxin-containing chow, spontaneous remyelination occurs. While this model does not recapitulate immune-mediated aspects of MS, it does allow for the investigation of fundamental mechanistic questions of the demyelination/remyelination process and roles of myelin in the stability of axonal domains^[75]^. The EAE model is an immune-mediated model that is induced through subcutaneous injection of myelin proteins accompanied by pertussis toxin and an adjuvant to ignite an inflammatory response^[75,79,80]^. The resulting neuroinflammation recapitulates key pathological features of MS such as inflammation, demyelination, and neuronal insults^[75,80,81]^. These two models allow for the rigorous assessment of MS-associated alterations in microglial-axonal interaction due to demyelination both in the presence of and independent from the autoreactive inflammatory response.

## MICROGLIAL CONTACT WITH THE NOR IN MODELS OF MS

Axonal function requires maintenance of the NOR^[82]^, and a major regulator of nodal axonal domain stability is myelin integrity^[77,83–89]^. For example, cuprizone-induced demyelination resulted in loss of nodal and paranodal clustered proteins^[77]^. Other studies have also demonstrated loss of nodal protein clustering as a downstream consequence of demyelination in mouse models of MS and postmortem MS tissue^[67,69,90,91]^. In addition to NOR disruption, analyses of human MS tissues have revealed that prominent microglia/macrophage accumulation correlates with active demyelination^[56,59,60,67]^. Indeed, myelin is required for NOR stability; however, NOR protein clustering can also be disrupted independent of demyelination. Howell *et al.*^[67]^ used immunohistochemical techniques to study NOR integrity in normal-appearing white matter of MS cases and in EAE and found NOR disruption correlated with local microglial inflammation but was independent of demyelinating lesions and did not correlate with the density of infiltrating lymphocytes. This was consistent with other studies demonstrating that numbers of microglia/macrophages correlate to EAE severity^[27,72–74]^. However, the cellular mechanisms by which microglia/infiltrating macrophages promote disease progression and whether these cells play differential roles in initiating demyelination or promoting repair remain unknown^[61,92,93]^. Yamasaki *et al.*^[6]^ began to elucidate the roles these cell types play in the disease course of EAE and their differential roles in myelin disruption. Serial block-face scanning electron microscopy of mice, in which the resident microglia fluoresced green and the infiltrating monocyte-derived macrophages fluoresced red, was utilized to distinguish the two inflammatory cell populations and to investigate their role in demyelination^[6]^. It was demonstrated that both microglia and infiltrating peripheral monocyte-derived macrophages contact the axo-glial unit at the NOR in the spinal cord of EAE-induced mice at disease onset [Figure 1B]^[6]^. They found that most (73%) of the NOR investigated (both intact and disrupted) were physically contacted by some sort of macrophage^[6]^. Interestingly, microglial association with the axo-glial unit was limited, while monocyte-derived infiltrating macrophage contact at the NOR was more extensive^[6]^. Monocyte-derived macrophage processes were found extended between the myelin and axolemma, potentially uprooting paranodal contacts and initiating demyelination [Figure 1B]^[6]^. In contrast, microglial processes contacted the axo-glial unit at the NOR, but the microglial processes did not extend beneath the axolemma and, instead, appeared to primarily interact with adjacent macrophages and appeared to be involved in debris clearance [Figure 1B]^[6]^. Gene expression profiles supported that infiltrating monocyte-derived macrophages were highly phagocytic and pro-inflammatory, whereas microglia demonstrated a suppressed cellular metabolism and activation phenotype^[6]^. These findings suggest that, at disease onset, infiltrating macrophages initiate active demyelination while microglia perform myelin debris clearance, a function that supports tissue regeneration and affects the maturation of oligodendrocyte progenitor cells^[3]^.

The differential mechanisms underlying microglial contact at the NOR is still to be fully determined. It was shown that C-C chemokine receptor type 2 (CCR2), a chemokine receptor essential for monocyte recruitment to CNS tissues during immune-mediated inflammation^[94,95]^, was important for recognition of disrupted NOR by infiltrating monocyte-derived macrophages^[6]^. Mice lacking CCR2 demonstrated reduced NOR contact by monocyte-derived macrophages and significantly less demyelination at EAE onset. Interestingly, CCR2-deficient mice displayed similar nodal pathology during the pre-onset stage of EAE (post-EAE induction but prior to onset of motor clinical symptoms), suggesting that inflammatory nodal disruption could be reversible if monocyte-derived macrophages were prevented from initiating demyelination at those sites^[6]^.

## MICROGLIAL CONTACT WITH THE AIS

Microglia contact the AIS during normal development and throughout life, indicating that these cells likely play a role in the regulation of AIS structure and/or function in both the developing and mature CNS [Figure 1A]^[45]^. A recent study utilizing both EAE and cuprizone models of MS to assess MS-related axonal injury and their underlying mechanisms found that inflammatory microglia and/or Macrophages physically contact the AIS^[27]^. It was found that the AIS is a primary target in disease pathogenesis of EAE^[27]^. In this study, mice were induced with either myelin-oligodendrocyte glycoprotein + EAE or cuprizone and AIS integrity of cortical neurons was assessed using immunohistochemical techniques. The integrity of the AIS was assessed by immunolabeling for ankyrinG (AnkG), a protein critical for AIS establishment and maintenance^[96–98]^. Upon EAE induction, it was found that the number and length of AISs were significantly reduced and that the number of disrupted AISs was associated with disease severity and progression^[27]^. This loss of AIS integrity, however, was not associated with demyelination, neuronal death, or axonal damage, rather appeared to be mediated by inflammatory factors^[27–29]^. Specifically, AIS disruption was preceded by microglial morphological changes suggestive of enhanced reactivity and increased contact by Iba-1 positive inflammatory cells but occurred independently of demyelination^[27]^. The nature of microglial interaction with the AIS changed substantially following EAE, transitioning from microglial process alignment along the AIS and periodic process ends contacting the AIS [Figure 1A] to microglial processes completely wrapping around the AIS [Figure 1B]^[27,99]^. Treatment with anti-inflammatory Didox, a free-radical scavenger and NF-κB modulator^[100–102]^, resulted in enhanced AIS structural integrity and reduction in microglial-AIS contact, indicating that EAE-induced inflammation is the driver for AIS disruption and enhanced microglial-AIS contact.

Microglial-AIS contact increased prior to and concomitant with changes in AIS structure, although it does not appear that contact alone drives AIS disruption. In the cuprizone model, demyelination and inflammation are present in the cortex; however, AISs were spared, suggesting the AIS, unlike the NOR, is not maintained by myelin presence^[27,103]^. Interestingly, in the cortex of cuprizone-fed mice, reactive microglia also enhanced contact with AISs but AIS structure was preserved^[27]^. Thus, the consequence of microglial-AIS contact appears to be stimulus dependent. In other models, microglia are recruited to and make contact with the initial portion of the axon and soma of hyperexcitable cells^[35,36]^. Microglial-axonal contact is activity dependent and results in a protective phenotype, preventing the neuron from excitotoxic death^[35,36]^. While live-imaging and physiological experiments have not been performed in MS models, analysis of AIS plasticity in EAE revealed structural changes of the AIS, such as decreased length^[27]^, which can occur in response to hyperexcitable environments^[104–106]^. Thus, the nature of microglial-AIS contacts may be context dependent and could either drive disruption or confer protection. Since the AIS is the axonal domain where action potentials are generated, this consistent microglial-AIS contact in both health and disease strongly implicates microglia as a regulator and/or modulator of neuronal function and further studies are needed to investigate the role of enhanced microglial interactions with the AIS in MS and its models.

The mechanisms mediating microglial contact with either the NOR or AIS remain undefined; however, as the molecular architecture is highly conserved between these two axonal segments, it is likely that the molecular mechanisms involved in associations with either region are similar. The fractalkine receptor CX_3_CR_1_ mediates microglial synaptic pruning and microglial contact with neuronal somatic-dendritic domains, and was, therefore, a prime candidate for mediating microglial-AIS contact^[107–109]^. However, absence of CX_3_CR_1_ fractalkine receptors on microglia did not alter contact with the AIS in the healthy mouse brain, suggesting that microglial-AIS interactions are not mediated through the fractalkine receptor^[45]^. Loss of brevican and versican, specialized extracellular matrix molecules surrounding the AIS, also did not alter microglial contacts onto the AIS^[45]^. In an effort to determine if AIS proteins are necessary for microglial contact, the AIS master scaffolding protein AnkG was knocked down, which disrupted AIS protein clustering and significantly reduced the number of microglial-AIS contacts, suggesting that molecules normally restricted to the AIS are important for microglial-AIS contact^[45]^. Thus, some progress has been made in eliminating candidates that mediate microglial-AIS contact and in determining that an intact AIS is important for microglial contact, but these experiments^[45]^ focused on microglial contact specifically with the AIS.

## NEUROINFLAMMATION IN TBI

TBI affects millions of people and is associated with devastating financial and societal costs linked to the long-term morbidities that develop and persist for years after the initial insult^[110–113]^. Recent studies have demonstrated the impact of inflammatory cascades in regulating many of these TBI-mediated outcomes^[114–118]^. While astrocytes and infiltrating peripheral monocytes/macrophages do play a role in TBI-induced neuroinflammation, microglia are thought to be the critical mediators of these TBI-induced neuroinflammatory processes and, therefore, have been the primary focus of TBI-related neuroinflammatory investigations. However, as it is difficult to specifically identify resident microglia from peripheral infiltrating monocytes following TBI, many studies call both populations “microglia” for simplicity. In the following sections, we do the same unless the population is specifically known to be infiltrating monocytic in origin.

Studies have also demonstrated neuroinflammation in various brain regions within the human population following TBI^[119–122]^. Molecular imaging studies have demonstrated microglial activation in populations of TBI patients as visualized via positron emission tomography using ligands for the mitochondrial translocator protein, TSPO, following brain injury^[114,119–121]^. While the TSPO ligands used in these studies have been shown to significantly increase binding to activated microglia post-TBI, they also bind to other neuroinflammatory cells following trauma^[114,119–121]^. Complementary histopathological studies investigating the extent and localization of various neuroinflammatory makers, including microglial CD68 and/or complement receptors, as well as morphological indications of microglial activation also demonstrated significant inflammation following brain injury in humans^[123–125]^. Many of these studies also indicate that neuroinflammation persists and evolves years after the initial head injury and that inflammation may become more severe with time post-injury^[117,121,125,126]^.

The majority of preclinical TBI models can be divided into focal and diffuse injury models, with some of the most used models being the controlled cortical impact (focal), central fluid percussion injury (diffuse), lateral fluid percussion injury (mixed focal and diffuse), and head rotational (diffuse) models; however, the specific models used to induce TBI are highly varied. For a review of the different types of TBI preclinical models, please see^[127,128]^. While the occurrence of microglial activation following TBI is rather well accepted, the role of activated microglia in the post-injury brain is far more enigmatic. A wide range of studies using various rodent models of brain injury have demonstrated that activated microglia can have a host of functions. For simplicity’s sake, these functions were lumped into two historical categories: M1, or pro-inflammatory microglia, that were involved with cytokine release that lead primarily to neuronal damage and M2, or anti-inflammatory microglia, that were associated with release of neurotrophic factors and cytokines downregulating the inflammatory responses^[129–132]^. These binary definitions, however, appear too simplistic for the complex interactions between the pro- and anti-inflammatory signals coming from activated microglia following TBI^[133]^. While the nomenclature for microglia falling along the inflammatory spectrum is still up for debate, studies do indicate that location, time following TBI, and systemic factors, including stress and infection, can push activated microglia toward a more pro-inflammatory state^[131,132,134,135]^. Information regarding these microglial populations is covered in greater detail in the following reviews^[129,133,134]^.

Many well-designed studies using rodents have indicated that reduction of activated microglial and/or targeting various neuroinflammatory signaling pathways ameliorates downstream pathology and behavioral morbidity^[136–149]^. One of the most common compounds used to assess the role of microglial activation following TBI is the second generation tetracycline drug, minocycline^[129]^. Minocycline is traditionally used clinically as an antibiotic; however, it has various other uses/effects including as a powerful anti-inflammatory compound^[140]^. Various studies demonstrate significant reductions in damaged or dying neurons, reduced lesion volumes, enhanced behavioral scores, and drastic reduction in pro-inflammatory cytokine expression following administration of minocycline, indicating that interactions between activated microglia and neurons could precipitate neurodegeneration^[141–143]^. In fact, minocycline is currently being assessed for safety in clinical trials for the treatment of TBI-associated morbidities thought to be regulated by inflammation^[144]^. However, other studies indicate that prolonged microglial inhibition via minocycline administration precipitates enhanced neurodegeneration and inflammation or no effect at all, demonstrating the complexity of neuroinflammatory responses following TBI^[145–147]^. Based on the fact that minocycline has a multitude of effects, it is also possible that the variability in these studies’ findings highlight the potential that non-inflammatory minocycline-induced reductions in TBI-mediated pathology in turn reduce inflammation and microglial activation^[140,147,148]^. In support of this possibility are studies showing little or no effect of genetic microglial elimination or direct microglial inhibition using compounds targeting the CSF1 receptor in altering TBI-induced pathology^[135,149,150]^. Additionally, administration of pro-inflammatory stimuli into the ventricle, surpassing induction of peripheral inflammatory responses, does not result in enhanced post-injury neurodegeneration, indicating that the peripheral inflammatory response, more than direct microglial activation, precipitates proinflammation-mediated secondary insults^[134,151]^. Overall, these studies underscore the intricacies of TBI-induced microglial activation and our limited understanding of microglial-neuronal interactions following brain injury.

## PHAGOCYTOSIS FOLLOWING TBI

One of the most well-studied physical interactions between microglia and neuronal segments following TBI is phagocytic engulfment. As in the non-injured brain, activated microglia serve a prominent and vital role in the clearance of cellular debris following brain injury. Upon the initial TBI insult, a multitude of cellular pathologies progress. One of the most well studied pathologies, and the hallmark of diffuse brain injury following TBI, is diffuse axonal injury/traumatic axonal injury^[125,152–156]^. Axonal injury first manifests as disruption of molecular transport anterogradely down the axon and progresses over hours, days, and months following injury to a disconnection at the point of initial transport disruption, resulting in a proximal axonal segment that remains connected to the neuronal cell body and a distal axonal segment that undergoes Wallerian degeneration^[157–159]^. Phagocytosis by activated microglia is required to engulf and clear away the axonal and myelin debris from the Wallerian degeneration of the distal axonal segment and involves the toll-like receptors, TREM-2, complement receptors 3 and 4, as well as MAC-2, for the engulfment of myelin, and the purinergic receptor P2RY6 [Figure 1C]^[160,161]^. Ultrastructural assessments of the injured brain have demonstrated significant phagocytosis of Wallerian debris by activated microglia following TBI^[52,116,162]^. Microglia with ameboid morphologies, indicative of phagocytic activity, were found primarily in proximity to the distal axonal segment sustaining dieback, but not the proximal axonal segments, following TBI-induced optic nerve damage^[163]^. Further, expression of mRNA indicative of phagocytic activity is significantly increased following trauma^[150]^. It should be noted, however, that both microglia and astrocytes containing phagocytic material have been observed, demonstrating that, while microglia may be the primary phagocytic cells in the brain, astrocytes also phagocytosis debris following injury^[162]^. Additionally, not all activated microglia were observed to be phagocytic following TBI, indicating that phagocytosis is not the only microglial-axonal interaction upregulated following TBI^[116]^.

## ROD MICROGLIA AND TBI

The readily identifiable, yet mysterious, “rod microglia” have been noted following TBI in a variety of pre-clinical models and in the human population. This subset of microglia appear following injury and are defined exclusively by their rod-like morphology and chain-like associations that form long microglial trains of several rod microglia lined up end-to-end [Figure 1C]^[164]^. These rod-shaped microglia have been described following a variety of neurological diseases, including neurosyphilis, and appear to be both non-phagocytic and reversible^[165]^. Both rod microglia and microglial trains appear primarily in brain regions in which the fiber tracks are linear, such as the neocortex, brainstem, and hippocampus^[124,166–168]^. This subset of rod microglia, however, appear to be absent in areas that are not linearly arranged, such as the thalamus^[166]^. The formation of microglial trains appears to be associated with p38; however the function of these microglial trains remains unknown^[169]^. Recently, it was found that microglial trains formed by rod microglia align with the apical dendrite, but not the axon as was previously thought, of pyramidal neurons in the rodent cortex [Figure 1C] and spatially associate with astrocytes, indicating that this subset of microglial-neuronal interaction is neuronal-segment specific and could be involved in an additional interplay between neuroinflammatory cell types^[150]^. However, the study of rod microglia following TBI is still in its infancy and requires further investigation into the timing and function of this microglial-axonal interaction subtype.

## TBI-INDUCED PROCESS CONVERGING AND DIVERGING MICROGLIA

**Figure 2.**
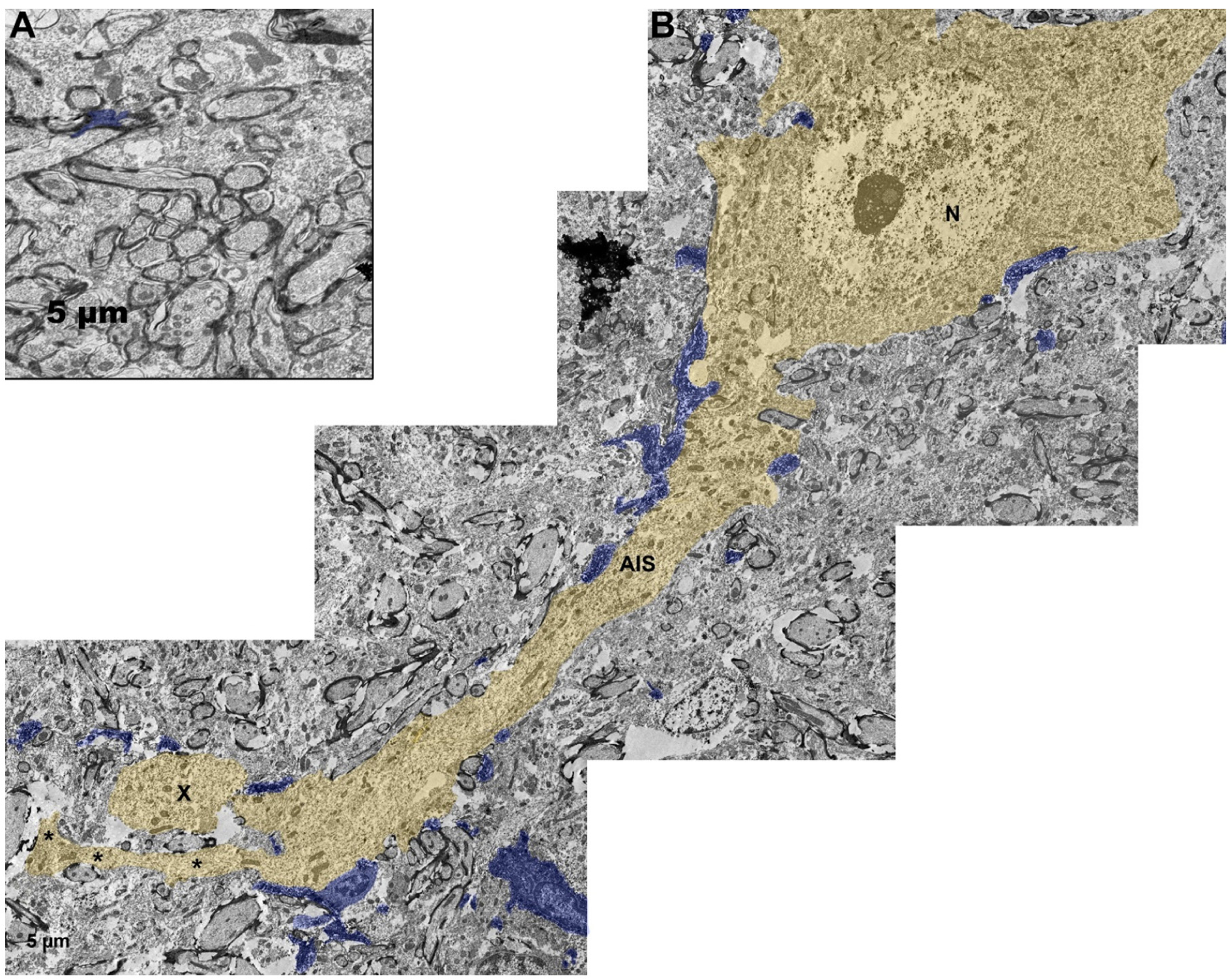
Ultrastructure of microglial process contacts onto axonal segments following TBI. Electron micrographs of Iba-1 immuno-labeled microglia contacting: A: intact non-injured axons in sham-injured micro pig thalamus; B: injured axonal segment in the thalamus of micro pigs acutely (one day) following diffuse TBI. Iba-1-labeled microglial processes are pseudo-colored blue and the injured axon is pseudo-colored yellow for clarity. While few microglial processes were observed in direct contact with axons normally, microglial processes were observed in direct contact various segments of the neuron, including the soma (N = nucleus), AIS, and the proximal axonal swelling (X) of the injured neuron. Note that the proximal axonal segment of the injured neuron demonstrates ultrastructural characteristics of axonal sprouting (*). Scale bar: 5 μm. AIS: axon initial segment; TBI: traumatic brain injury

Over the last several years, another subtype of microglial-neuronal interaction has been observed following brain injury. This interaction subtype manifests as physical contacts between activated microglia and the proximal axonal segment of injured axons following TBI^[51]^. Using a micro pig model of central fluid percussion-generated diffuse TBI, paired with multiplexed immunohistochemical quantitative image analysis, recent studies found processes from activated microglia converge onto adjacent injured thalamic axons acutely (hours to one day post-injury) following injury, a phenomenon termed “microglial process convergence” (MPC; Figure 1C)^[50,52]^. These process converging (PC) microglia were neither ameboid nor rod shaped, rather they displayed shortened processes, with fewer process branches, morphological changes indicative of activation without progression to phagocytosis^[13,51,52]^. Ultrastructural assessments confirmed that PC microglia were non-phagocytic in nature^[50,52]^. Additionally, a single injured axon could have processes converging from multiple PC microglia^[50,52]^. As the majority of diffuse axonal injury following TBI appears to occur in or adjacent to the AIS, it is likely that the proximal axonal swellings in which this subtype of PC microglia are converging are nearer to the AIS than to more distal points along the axon [Figure 2]^[170,171]^. Another group using a micro pig model of head-rotation-induced diffuse TBI found indications of potential PC microglia associated with injured neurons following brain injury^[172]^. Specifically, also using multiplexed immunohistochemical quantitative image analysis, they observed that microglia were in closer proximity to injured neuronal soma in multiple brain regions following TBI compared to neurons in sham injured micro pigs [Figure 1C]^[172]^. These PC microglia also appeared activated without falling into the morphological categories of phagocytic or rod microglia^[172]^. Another recent study found that MPC onto cell bodies of injured neurons is associated with protection. Specifically inhibition of this MPC increased ischemia-induced lesion volume, behavioral morbidity, and calcium influx^[37]^. Additionally, ischemic injury results in microglial process contacts with injured synapses that are nearly 10 times longer in duration than the 4–5-min-long contacts observed in non-injured animals using a thinned-skull live imaging approach^[9]^. Therefore, it is likely that MPC could involve an increase in both the number of microglial processes as well as the duration of these contacts onto injured axons.

The mechanisms involved in regulating MPC onto neuronal and axonal segments has primarily been studied in mouse models of epilepsy. The number of microglial process contacts appear to be directly related to the level of neuronal activity, in that MPC was significantly reduced upon reduction in neuronal activity via either temperature reduction or tetrodotoxin administration in thinned-skull live-imaging studies^[9]^. Induction of neuronal hyperexcitability to the point of excitotoxicity also promoted MPC^[173]^. Hyperexcitability-induced MPC resulted in reduced neuronal activity and overall increased neuronal survival in the face of otherwise excitotoxic events that were not seen following microglia elimination or inhibition of MPC^[173,174]^. Neuronal excitation precipitates higher extracellular and lower intracellular Ca^2+^ concentrations and increased extracellular ATP concentrations around the active neuron, which appear to be primary molecular mediators of hyperexcitability-induced MPC^[173–176]^. ATP-mediated MPC was found to promote polarization of microglial process outgrowth toward the location with high ATP levels [Figure 1C]^[177]^. Elimination of the purinergic receptor P2Y12 or the fractalkine receptor CX3CR1 drastically reduced MPC onto hyper-excitable neurons, indicating that microglial P2Y12 and CX3CR1 are required for ATP-mediated MPC^[176,178]^. Excitatory neurons also release glutamate upon excitation. Concentration of extracellular glutamate has also been found to mediate hyperexcitability-induced MPC potentially via activation of N-methyl-D-aspartate (NMDA) receptors^[174,178]^. Glutamate/NMDA-mediated MPC was also found to require microglial P2Y12, but not CX3CR1^[177]^. Glutamate-mediated MPC also promoted nonpolarized outgrowth of microglial processes, indicating that different molecular mechanisms of hyperexcitability-induced MPC may result in different forms of MPC [Figure 1C]^[177]^. Further, these mechanisms appear distinct from those involved in microglial phagocytosis, as knocking out or inhibiting P2Y12 or NMDA inhibited MPC without affecting phagocytosis^[178]^. While epilepsy and TBI are different CNS diseases with distinct neuropathologies, the molecules and mechanisms discussed above are prime candidates for regulation of TBI-induced MPC. In fact, the Jacobs group found that both axotomized and intact neurons demonstrate hyperexcitability one day following TBI in mice that appears to resolve in the axotomized population, but not the intact neurons, by two days post-injury^[179,180]^. While TBI-induced MPC onto the proximal axonal segments of axotomized neurons has yet to be thoroughly investigated, these findings indicate the potential that similar mechanisms might be at play in TBI and epilepsy-induced MPC.

It appears that TBI-induced MPC may be species dependent, as it was found that rats sustaining the same central fluid percussion injury paradigm as their pig counterparts did not demonstrate MPC^[50]^. Rather, at the same time points following injury, there was a significant decrease in microglial contacts onto injured proximal axonal segments in the rats, indicating microglial processes that diverged from injured axons or microglia process divergence (MPD)^[50]^. This MPD observed in rats is in alignment with previous observations in injured rats and mice that activated microglia do not physically associate with proximal segments of injured axons following brain injury^[116,163]^. TBI-associated MPD was also observed by a group assessing the occurrence of microglia associations with the AIS, regardless of axonal injury, following TBI in mice^[45]^. They demonstrated that microglial contacts onto the AIS of axons significantly decreased following TBI in mice, indicating MPD similar to that observed by the other groups following TBI in rodents^[45,50,116,163]^. In contrast to those studies, however, these AIS-associating, or “AXIS”, microglia were not specific to injured axonal segments and appeared ramified (morphologically not activated), indicating that these AXIS microglia could represent a distinct subtype of MPD microglia^[45,50]^. The interaction between the AXIS microglia and the AIS appears to be ankrin-G and GABA mediated, while the fractalkine receptor, CX3CR1, does not appear necessary for the AXIS microglial interactions^[45]^.

There are reports indicating that microglia physically interact with injured axons following TBI in the human brain. In 2014, a study demonstrated co-labeling of microglia with injured proximal axonal swellings in brains of veterans who had histories of blast injury exposure^[181]^. Another study showed potential PC microglia contacting injured axonal swellings when employing double-labeling techniques in human TBI tissue^[182]^. These studies indicate that microglial processes may contact axonal swellings in the human brain following TBI; however, further investigation is needed to comprehensively assess potential alterations in microglial-neuronal physical interactions in the human population and address how those changes compare to those observed pre-clinically.

Additionally, a study investigating the expression of neuronal outgrowth marker, GAP43, in injured axonal segments as it related to the density of microglia in brain tissue from people diagnosed with MS or TBI demonstrated a positive correlation between neuronal regeneration and microglial density following TBI in clinical samples [Figure 1C]^[183]^. Other studies have also observed GAP43 expression in proximal axonal swellings following injury in both human tissue and following induction of TBI in pre-clinical models^[184–186]^. Ultrastructural assessments further demonstrated morphological alterations indicative of active axonal sprouting of proximal axonal swellings following TBI, demonstrating that axonal process outgrowth following TBI is possible and potentially likely [Figure 2]^[184,185]^. Microglia have been shown to express neurotrophic factors, such as nerve growth factor, following TBI, supporting a potential role for MPC in post-injury axonal outgrowth^[187]^. Microglia may also release exosomes that induce neurite outgrowth^[19]^. The role of MPC and/or MPD in potential post-injury axonal sprouting, however, remains speculative.

## CONCLUSION

It is well accepted that microglia mediate neuroinflammatory processes in health and disease via proand anti-inflammatory cytokines and chemokines. However, microglia also appear to mediate neuronal function through physical contacts onto various neuronal segments, including dendrites, synapses, cells bodies, and axons. While the study of microglial-axonal contacts is still in its infancy, there are indications that these contacts play diverse and important roles during normal development and in the healthy CNS as well as following TBI or in disease states, such as MS. Analysis of microglia in experimental and human tissues demonstrate that microglia exhibit a spectrum of morphologies including ramified, rod-like, hypertrophied, and ameboid that all exert unique contact subtypes onto axonal segments indicative of the diverse roles microglial-axonal interactions play. Microglia and infiltrating monocytes contact various axonal segments in unique and specific ways that appear tied to the axonal region contacted, the morphology of the microglia, and the disease state. Further, it appears that the presence of microglia contacts at axonal domains may confer protection. Some of the immediate questions for this burgeoning field focus on the potential ameliorative effects of microglial contacts onto axonal and other neuronal segments as well as the timing of these interactions following various pathologies. Future examinations of axonal interactions using functional assessments and live imaging techniques could refine the distinction between axonal contacts of resident microglia and those formed by peripheral monocyte-derived infiltrating macrophages and help elucidate the nature of these interactions. Furthermore, identifying the molecules mediating contact between microglia and the axon will point toward new strategies to treat disease and promote repair in diverse inflammatory pathologies. The studies reviewed herein underscore the importance of microglial-axonal contacts in the regulation of neural signaling and the need for further investigation into these variable interactions in both the healthy and injured CNS.
